# HALT-D: a randomized open-label phase II study of crofelemer for the prevention of chemotherapy-induced diarrhea in patients with HER2-positive breast cancer receiving trastuzumab, pertuzumab, and a taxane

**DOI:** 10.1007/s10549-022-06743-9

**Published:** 2022-10-25

**Authors:** Paula R. Pohlmann, Deena Graham, Tianmin Wu, Yvonne Ottaviano, Mahsa Mohebtash, Shweta Kurian, Donna McNamara, Filipa Lynce, Robert Warren, Asma Dilawari, Suman Rao, Candace Mainor, Nicole Swanson, Ming Tan, Claudine Isaacs, Sandra M. Swain

**Affiliations:** 1 Georgetown Lombardi Comprehensive Cancer Center, Washington, DC USA; 2 MedStar Georgetown University Hospital, Washington, DC USA; 3 Department of Breast Medical Oncology, University of Texas MD Anderson Cancer Center, Houston, TX USA; 4 Hackensack University Medical Center, Hackensack, NJ USA; 5 Clinical Research Management Office, Georgetown University Medical Center, Washington, DC USA; 6 Medstar Franklin Square Medical Center, Baltimore, MD USA; 7 Dana-Farber Cancer Institute, Boston, MA USA; 8 Present Address: FDA Center for Drug Evaluation and Research, Silver Spring, MD USA; 9 Department of Biostatistics, Bioinformatics, and Biomathematics, Georgetown University Medical Center, Washington, DC USA; 10 MedStar Health, Washington, DC USA

**Keywords:** Trastuzumab, Pertuzumab, Chemotherapy-induced diarrhea, Taxane, Crofelemer, HER2-positive breast cancer

## Abstract

**Purpose:**

To assess whether crofelemer would prevent chemotherapy-induced diarrhea (CID) diarrhea in patients with HER2-positive, any-stage breast cancer receiving trastuzumab (H), pertuzumab (P), and a taxane (T; docetaxel or paclitaxel), with/without carboplatin (C; always combined with docetaxel rather than paclitaxel).

**Methods:**

Patients scheduled to receive ≥ 3 consecutive TCHP/THP cycles were randomized to crofelemer 125 mg orally twice daily during chemotherapy cycles 1 and 2 or no scheduled prophylactic medication (control). All received standard breakthrough antidiarrheal medication (BTAD) as needed. The primary endpoint was incidence of any-grade CID for ≥ 2 consecutive days. Secondary endpoints were incidence of all-grade and grade 3/4 CID by cycle/stratum; time to onset and duration of CID; stool consistency; use of BTAD; and quality of life (Functional Assessment of Chronic Illness Therapy for Patients With Diarrhea [FACIT-D] score).

**Results:**

Fifty-one patients were randomized to crofelemer (*n* = 26) or control (*n* = 25). There was no statistically significant difference between arms for the primary endpoint; however, incidence of grade ≥ 2 CID was reduced with crofelemer vs control (19.2% vs 24.0% in cycle 1; 8.0% vs 39.1%, in cycle 2). Patients receiving crofelemer were 1.8 times more likely to see their diarrhea resolved and had less frequent watery diarrhea.

**Conclusion:**

Despite the choice of primary endpoint being insensitive, crofelemer reduced the incidence and severity of CID in patients with HER2-positive breast cancer receiving P-based therapy. These data are supportive of further testing of crofelemer in CID.

**Trial registration:**

Clinicaltrials.gov, NCT02910219, prospectively registered September 21, 2016.

**Supplementary Information:**

The online version contains supplementary material available at 10.1007/s10549-022-06743-9.

## Introduction

Chemotherapy-induced diarrhea (CID) is debilitating, with a detrimental impact on quality of life [1, 2]. It occurs in ≤  ~ 80% of patients with breast cancer (BC) receiving trastuzumab and pertuzumab (HP; F. Hoffmann-La Roche Ltd, Basel, Switzerland/Genentech, Inc., South San Francisco, CA, USA), plus a taxane (T) [3, 4]. This reaches grade 3 in ~ 8–12% [5–8]. Various antidiarrheal agents are available for symptom management [2]; however, none target the underlying mechanism. This is predominantly secretory diarrhea from excess chloride ion and fluid secretion in the intestinal lumen through activation of apical cystic fibrosis transmembrane conductance regulator (CFTR) or calcium-activated chloride channels (CaCC) [9]. CID in HP-containing regimens may be caused by EGFR downregulation and/or blockade, which leads to excess chloride secretion and secretory diarrhea through reversal of the acute inhibitory effect of epidermal growth factor on chloride secretion [10–12]. CFTR/CaCC activation by H/P has not been described; however, EGFR inhibition-related CFTR/CaCC-mediated chloride ion secretion [12–15] would apply to H and P, both of which are associated with diarrhea [5]. Enterocyte apoptosis, impaired regeneration, and the chloride ion-mediated secretory mechanism of diarrhea that occurs with EFGR inhibitors may explain why onset with EGFR inhibitors is not immediate and may worsen over time. This is especially relevant when HP is added to agents that are directly toxic to gastrointestinal cells.

Crofelemer (Napo Pharmaceuticals, Inc., San Francisco, CA, USA) is a novel oral botanical antisecretory, antidiarrheal drug purified from *Croton lechleri* tree sap [16]. Crofelemer regulates luminal chloride efflux and fluid secretion through the use-dependent inhibition of CFTR and CaCC chloride ion channels in the apical membrane of the intestinal mucosa [17] and is FDA-approved for adult patients with HIV with non-infectious diarrhea receiving antiretroviral therapy [18]. Due to its large molecular size and polarity, crofelemer acts mainly in the lumen and, thus, is typically well tolerated [17, 19–21]. Only a negligible amount is systemically absorbed following oral dosing in humans in the fasted or fed state [22]. Since crofelemer is the only known selective and specific use-dependent inhibitory modulator of CFTR and CaCC [17], the HALT-D study (NCT02910219) evaluated prophylaxis of diarrhea with crofelemer in patients with BC receiving HP-containing regimens. However, diarrhea remains a challenging endpoint for clinical trials as it is difficult to measure [23] (Version 4.0 of the National Cancer Institute’s Common Terminology Criteria for Adverse Events [NCI-CTCAE] merely defines diarrhea as a disorder characterized by frequent and watery bowel movements [24]). Recall bias, time to onset, frequency of bowel movements, frequency of watery stools, diarrhea incidence, diarrhea duration, repeated measures, clustering of events, and clinical impact of diarrhea are characteristics that may interfere with overall evaluation.

In HALT-D, we hypothesized that crofelemer would reduce diarrhea in patients with HER2-positive BC receiving HP and a taxane with or without carboplatin (THP/TCHP; the taxane being paclitaxel or docetaxel; where C was always combined with docetaxel rather than paclitaxel) in the neoadjuvant, adjuvant, or metastatic settings. When HALT-D was planned, there was, and continues to be, no established gold standard endpoint for assessing diarrhea in clinical trials. An expert group published recommendations in 2004, but acknowledged that standard practices for assessment and management are needed [25]. The Bristol Stool Form Scale (BSFS) [26, 27] provides one assessment method, but there is inherent subjectivity in classification, and disparities in endpoints and regulatory guidance that require further investigation in large clinical trials [23].

In the absence of a gold standard endpoint, we selected the incidence of any-grade diarrhea for ≥ 2 consecutive days. Here, we present the primary analysis of HALT-D.

## Methods

### Oversight

HALT-D was conducted according to Good Clinical Practice and the principles of the Declaration of Helsinki. All patients provided written informed consent. Protocol approval was obtained from the Georgetown University Institutional Review Board. Safety data were reviewed semi-annually by an independent Data and Safety Monitoring Committee. Crofelemer was provided by Napo Pharmaceuticals, Inc.

### Patients

As previously described [28], eligible patients were ≥ 18 years with any-stage HER2-positive BC, scheduled to receive ≥ 3 consecutive THP/TCHP cycles, had an Eastern Cooperative Oncology Group performance status 0–2, and adequate organ function. Patients with irritable bowel syndrome, colitis, recent antibiotic use, active systemic infection, ostomy, prior total colectomy, fecal incontinence, abdominal radiation, and major abdominal or pelvic surgery within the past 6 months or without recovery of bowel function were excluded.

### Trial design and procedures

**Fig. 1:**
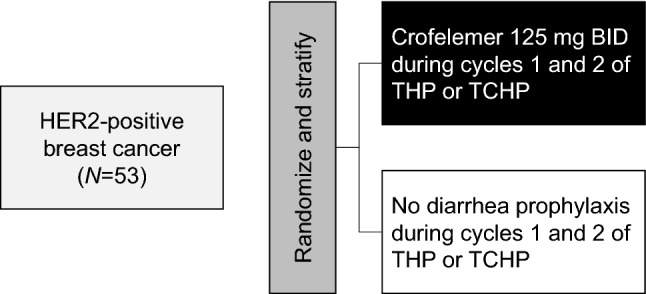
Study schema. *BID* twice daily, *C*, carboplatin; *H* trastuzumab, *P* pertuzumab, *T* taxane. Reprinted from Clinical Breast Cancer, 17, Gao JJ, Tan M, Pohlmann PR, and Swain SM, HALT-D: A Phase II Evaluation of Crofelemer for the Prevention and Prophylaxis of Diarrhea in Patients With Breast Cancer on Pertuzumab-Based Regimens, 76–78, Copyright (2016), with permission from Elsevier

The study schema and schedule of events are presented in Fig. 1 and Table 1.

**Table 1:** Schedule of events

	Treatment cycle	1			2			3			4
	Week	1	2	3	1	2	3	1	2	3	1
Treatment	Pertuzumab	X			X			X			X
	Trastuzumab	X			X			X			X
	Docetaxel or paclitaxel	X	X	X	X	X	X	X	X	X	X
	Carboplatin (if using)	X			X			X			X
	Crofelemer (or observation)	X	X	X	X	X	X				
Diaries	Crofelemer diary	X	X	X	X	X	X				
	Rescue medication diary	X	X	X	X	X	X	X	X	X	
	Bowel movement diary	X	X	X	X	X	X	X	X	X	
	Clinic visit (each Day 1)	X			X			X			X
	FACIT-D (each Day 1)	X			X			X			X

*FACIT-D* Functional Assessment of Chronic Illness Therapy for Patients With Diarrhea

Patients were observed on-study for three cycles. Chemotherapy/HER2-targeted therapy doses were paclitaxel 80 mg/m^2^ intravenously (IV) every week (q1w) or docetaxel 75 mg/m^2^ IV q3w; C area under the concentration–time curve 6 IV q3w; H 8 mg/kg IV loading dose, 6 mg/kg IV maintenance doses q3w; and P 840 mg IV loading, 420 mg IV maintenance q3w.

Patients were randomized 1:1 to crofelemer 125 mg delayed release tablets orally twice a day (BID, or no scheduled prophylactic medication in the control (observation) group during cycles 1 and 2 of chemotherapy/HER2-targeted therapy. Randomization was performed by statisticians in the Georgetown Lombardi Comprehensive Cancer Center Biostatistics and Bioinformatics Shared Resource (Washington, DC, USA) who also generated the randomization table and held the key. Stratification was by chemotherapy regimen (HP with paclitaxel, HP with docetaxel, or docetaxel with CHP). The first crofelemer dose was administered 30–60 min prior to the first chemotherapy/HER2-targeted therapy cycle. All other doses were taken at home. At the beginning of cycles 1 and 2, patients randomized to crofelemer received a diary to record administration date and time during each cycle. This was collected at the end of chemotherapy/HER2-targeted therapy cycles 1 and 2. There was no crofelemer administration during cycle 3 (empiric to use two cycles).

At the start of treatment, all patients received recommendations for standard-of-care breakthrough antidiarrheal medication (BTAD) as needed, with no scheduled antidiarrheal prophylactic medication. Patients also received instructions to record BTAD use in a diary: drug name, dose, and administration date/time during all cycles. The diary was collected at the end of cycles 1, 2, and 3.

Baseline daily number of bowel movements was documented before the start of treatment. Investigators considered all clinical data at each follow-up visit and prospectively documented diarrhea events per NCI-CTCAE v4.0. For patient-reported outcomes (PROs), by Day 1 of cycle 1 all patients received a BSFS illustrated form and bowel movement diary. Patients were instructed to record the date, time, and BSFS stool consistency of each movement during the three cycles. Watery diarrhea was defined as BSFS 6–7; non-watery, as 1–5 [26, 27], for both investigator-assessed outcomes and PROs. The probability of watery diarrhea was calculated by repeated logistic regression for each cycle. Diaries were collected by the study team at the end of each cycle (Table 1).

All patients filled out quality-of-life questionnaires based on Functional Assessment of Chronic Illness Therapy for Patients With Diarrhea (FACIT-D) scores on the first day of each cycle, as well as at the end of study participation.

### Statistics

The primary endpoint (incidence of any-grade diarrhea for ≥ 2 consecutive days) was assessed by NCI-CTCAE v4.0. Secondary endpoints included incidence of all-grade and grade 3–4 CID by cycle and by stratum; time to onset and duration of CID; stool consistency; frequency of BTAD use; adverse events by stratum; and FACIT-D total and diarrhea subset scores. If the primary endpoint was not met statistically, the secondary endpoints and overall incidence of all-grade diarrhea in both arms were still evaluated for clinical benefit. Patients were observed for adverse events during three cycles.

Fisher’s exact test was used for comparing binary and categorical variables, and summary statistics and the Wilcoxon test were used for ordinal grade/scale variables. The trial was designed to detect a 40% absolute decrease in incidence of CID (60% to 20%), with a two-sided significance level of 0.10. To analyze the time to onset of diarrhea (event), Log-rank tests were performed. Repeated measures logistic regression with computation by generalized estimating equations (SAS Proc GENMOD) was used to assess the overall probability of having watery diarrhea in each treatment cycle. Recurrent survival model of Prentice–Williams–Peterson for times to resolution of diarrhea was used to analyze duration of diarrhea within each cycle between arms, considering multiple bouts of diarrhea with different durations for one patient within a cycle. The likelihood ratio test (LRT) with an ordinal regression model was used to determine whether there was an interaction between crofelemer effect and chemotherapy regimen. Medians and percentage reductions in median value of watery diarrhea episodes per week were calculated comparing crofelemer versus control. The Wilcoxon rank sum test was conducted for two independent groups and the Wilcoxon signed rank test was conducted for paired data. Safety data are descriptive.

Anticipating a 10% withdrawal rate, enrollment of 52 patients was planned. Statistical analyses were performed using R 3.6.2 (R Foundation, Vienna, Austria) and SAS (SAS Institute, Cary, NC, USA).

## Results

Fifty-three patients were enrolled between 02/21/2017 and 08/25/2020 to crofelemer (*n* = 27) or control (*n* = 26). One withdrew consent prior to starting and another soon after receiving first dose of chemotherapy (no data collected). These patients were excluded from analysis; 26 and 25, respectively, were analyzed. A further 46 were pre-screened and excluded for reasons such as not meeting eligibility criteria, declining treatment, having already started treatment, and provider decision.

Early treatment discontinuation occurred in six cases: complications of diarrhea (*n* = 1, control group), chemotherapy regimen changed during study participation for causes other than diarrhea (*n* = 4), and non-compliance with trial procedures (*n* = 1).

**Fig. 2:**
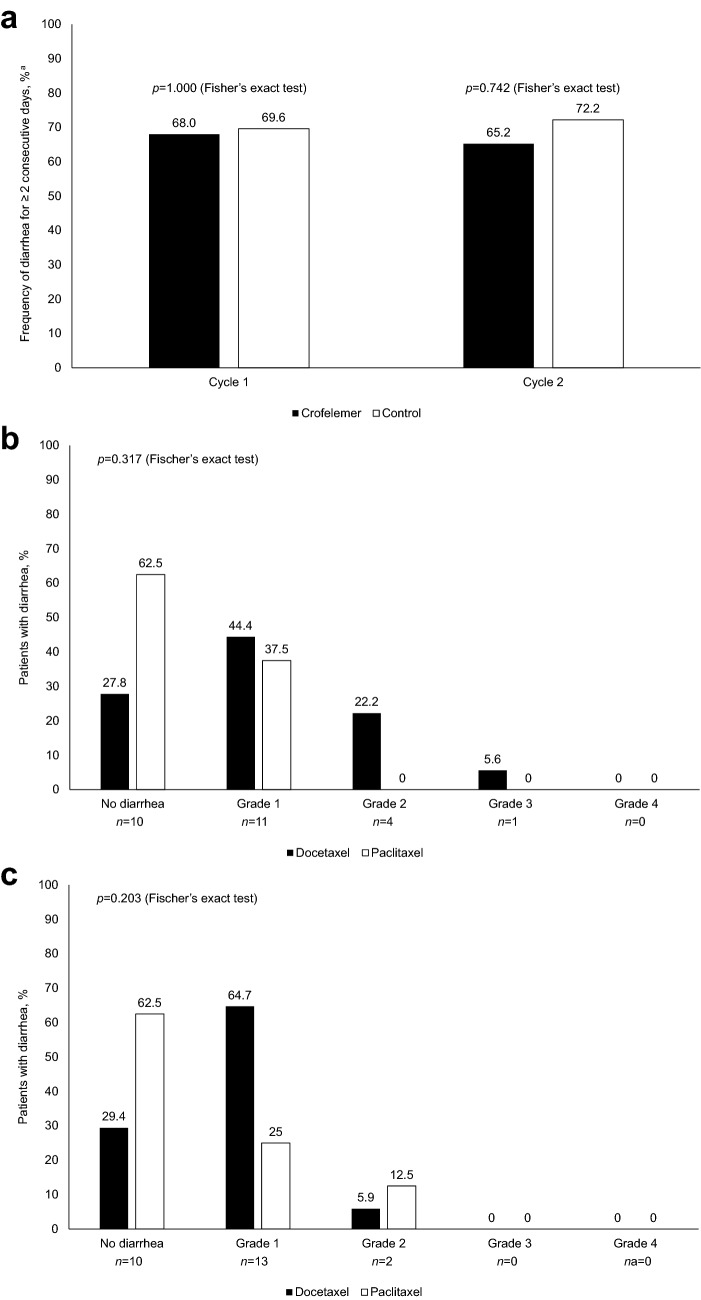
Frequency of diarrhea for ≥ 2 consecutive days (primary endpoint) (**a**); frequency of diarrhea according to taxane and treatment cycle (crofelemer arm only) in cycle 1 (**b**) and cycle 2 (**c**). ^a^Investigator-assessed

Demographics were well balanced between the two arms (Table 2). Rescue medications included diphenoxylate, diphenoxylate hydrochloride/atropine, diphenoxylate hydrochloride/atropine and loperamide, loperamide, and loperamide and octreotide acetate. Diarrhea was overall more frequent in cycle 1. The primary endpoint was similar between arms. During cycle 1, 68.0% and 69.6% of patients had diarrhea for ≥ 2 consecutive days in the crofelemer and control arms. During cycle 2, the numbers were 65.2% and 72.2% for the control arm (not statistically different: *p* = 0.742; Fig. 2A).

**Table 2:** Patient demographics

	Crofelemer (*n* = 26)	Control (*n* = 25)	Overall (*n* = 51)
Median age at diagnosis, years (range)	52.4 (37.0, 70.6)	50.2 (26.8, 66.0)	50.8 (26.8, 70.6)
Female gender (self-assigned)	26 (100)	25 (100)	51 (100)
Race, *n* (%)			
African American	9 (34.6)	6 (24.0)	15 (29.4)
Caucasian	14 (53.8)	17 (68.0)	31 (60.8)
Asian	1 (3.8)	0	1 (2.0)
Not reported/unknown	2 (7.7)	2 (8.0)	4 (7.8)
Mean weight, kg (SD)	77.7 (17.5)	81.3 (32.3)	79.4 (25.3)
Mean body surface area, m^2^ (SD)	1.87 (0.23)	1.89 (0.44)	1.88 (0.34)
ECOG PS			
0	19 (73.1)	19 (76.0)	38 (74.5)
1	2 (7.7)	0	2 (3.9)
2	0	1 (4.0)	1 (2.0)
Unknown	5 (19.2)	5 (20.0)	10 (19.6)
Cancer stage at diagnosis, *n* (%)			
I	3 (11.5)	5 (20.0)	8 (15.7)
II	12 (46.2)	7 (28.0)	19 (37.3)
III	5 (19.2)	9 (36.0)	14 (27.5)
IV	6 (23.1)	4 (16.0)	10 (19.6)
Treatment intent, *n* (%)			
Curative	14 (53.8)	14 (56.0)	28 (54.9)
Palliative	12 (46.2)	11 (44.0)	23 (45.1)
Treatment type, *n* (%)			
TCHP (docetaxel)	14 (53.8)	14 (56.0)	28 (54.9)
THP (docetaxel)	4 (15.4)	3 (12.0)	7 (13.7)
THP (paclitaxel)	8 (30.8)	8 (32.0)	16 (31.4)
Mean number of daily bowel movements at baseline (SD)	1.5 (0.653)	1.3 (0.703)	1.4 (0.679)

Data are number of patients, *n* (%), median (range), or mean (SD) where specified

All AEs shown were grade 1/2

*ECOG* PS Eastern Cooperative Oncology Group performance status

*AE* adverse event, *C* carboplatin, *H* trastuzumab, *P* pertuzumab, *SD* standard deviation, *T* taxane

Several secondary endpoints favored crofelemer (Table 3). Furthermore, in cycle 2, no patients in the crofelemer arm experienced grade 3–4 diarrhea (vs 17.3% in the control arm). Patients in the crofelemer arm experienced significantly less grade ≥ 2 CID than the control arm during cycle 2, based on PROs and investigator reporting (Table 4).

**Table 3:** Probability of watery diarrhea (Bristol Stool Form Scale 6–7) and CID resolution according to treatment arm

	Cycle	OR	95% CI	*p *value
Probability of watery diarrhea (any grade; crofelemer vs control)	1	0.77	0.6129, 0.9774	0.03^a^
	2	0.84	0.6491, 1.0805	0.17^a^
		HR	95% CI	
Probability of CID resolution	1	1.03	0.5928, 1.79	0.916^b^
	2	1.804	1.02, 3.189	0.0425^b^

^a^Repeated logistic regression by generalized estimating equations

^b^Recurrent survival model Prentice–Williams–Peterson

*CI* confidence interval, *CID* chemotherapy-induced diarrhea, *HR* hazard ratio, *OR* odds ratio

**Table 4:** Incidence of diarrhea according to NCI-CTCAE grade and treatment cycle

	Cycle	NCI-CTCAE v4.0 grade	Crofelemer		Control		*p* value (Wilcoxon rank sum test)
Maximum diarrhea grade (investigator assessed)	1		*n* = 26		*n* = 25		
		No diarrhea	10 (38.5)		9 (36.0)		
		1	11 (42.3)		10 (40.0)		
		2	4 (15.4)	Grade 2–4: 5 (19.2)	4 (16.0)	Grade 2–4: 6 (24.0)	0.7244
		3	1 (3.8)		2 (8.0)		
		4	0		0		
	2		*n* = 25		*n* = 23		
		No diarrhea	10 (40.0)		5 (21.7)		
		1	13 (52.0)		9 (39.1)		
		2	2 (8.0)	Grade 2–4: 2 (8.0)	5 (21.7)	Grade 2–4: 9 (39.1)	0.0196
		3	0		3 (13.0)		
		4	0		1 (4.3)		
Maximum diarrhea grade (PROs)	1		*n* = 26		*n* = 23		
		No diarrhea	2 (7.7)		2 (8.7)		
		1	18 (69.2)		9 (39.1)		
		2	4 (15.4)	Grade 2–4: 6 (23.1)	10 (43.5)	Grade 2–4: 12 (52.2)	0.1112
		3	2 (7.7)		2 (8.7)		
		4	0		0		
	2		*n* = 22		*n* = 18		
		No diarrhea	2 (9.1)		0		
		1	18 (81.8)		12 (66.7)		
		2	1 (4.5)	Grade 2–4: 2 (9.0)	4 (22.2)	Grade 2–4: 6 (33.3)	0.0361
		3	1 (4.5)		2 (11.1)		
		4	0		0		

Data are number of patients (%)

*NCI-CTCAE* National Cancer Institute’s Common Terminology Criteria for Adverse Events, *PROs* patient-reported outcomes

Watery diarrhea of any grade occurred less frequently in the crofelemer arm than in the control arm for cycle 1; the smaller difference between the groups was not statistically significant for cycle 2 (Table 3).

Use of rescue medication for treatment of emerging CID, time to onset, and duration of diarrhea after each cycle of chemotherapy/HER2-targeted therapy were not different between crofelemer and observation at cycle 1 or cycle 2 (data not shown), neither were FACIT-D scores (Online resource 1).

The probability of resolution of CID was evaluated by considering cases with ≥ 1 bout of diarrhea within the same chemotherapy cycle. There were 17/31 patients who had diarrhea in cycle 1 with recurring events (≥ 2 bouts; resolved and occurred again) during cycle 1. The hazard ratio (HR) for CID resolution in cycle 1 was 1.03. There were 19/27 patients who had diarrhea in cycle 2 with recurring diarrhea during cycle 2. The HR for CID resolution in cycle 2 was 1.804, meaning CID in patients in the crofelemer arm was ~ 1.8 times more likely to resolve than in the control arm during cycle 2 (Table 3).

Online resource 2 shows medians and percentage reductions in watery bowel movements from the control arm per week in cycles 1, 2, and 3. Patients in the crofelemer arm received crofelemer only in cycles 1 and 2 (standard of care was given in cycle 3 to all). During cycles 1 and 2, watery bowel movements were less frequent in the crofelemer arm compared with control; however, results were not statistically significant. When comparing cycle 3 with cycle 2 in the crofelemer arm only, medians suggested that patients had more diarrhea during cycle 3; again, results were not statistically significant.

Since randomization was stratified by chemotherapy regimen, potential interactions between crofelemer and regimen (docetaxel- vs paclitaxel-based) could be assessed. CID frequency in cycles 1 and 2 varied according to chemotherapy regimen in patients who received crofelemer (Fig. 2B and C). THP with paclitaxel was more often associated with no diarrhea than the docetaxel-containing regimens (THP or TCHP) but this was not statistically significant at cycles 1 or 2. LRTs of ordinal regression models showed no significant interaction between crofelemer usage and chemotherapy regimen during cycle 2 (LRT = 3.243, degrees of freedom = 1, *p* = 0.072).

Frequency of the most common non-diarrhea adverse events was similar in both arms (Online resource 3). During cycles 1 and 2, the most frequent were fatigue (*n* = 10 patients in the crofelemer arm and 9 in the control arm), nausea (*n* = 10 and 9), anemia (*n* = 5 and 2), anorexia (*n* = 4 and 3), mucositis oral (*n* = 4 and 3), and constipation (*n* = 4 and 2).

In the crofelemer arm, only one patient experienced a serious adverse event: grade 4 neutropenia attributed to chemotherapy (not crofelemer-related). In the control arm, seven patients experienced serious adverse events: port-a-cath infection, obstruction gastric, cellulitis, hypoglycemia, dehydration, neutropenia, hyperglycemia, hyperkalemia, acidosis, confusion, fatigue, urinary tract infection, chest pain—cardiac, atrial flutter, and fever (all single events and unrelated to treatment; one patient experienced eight events and one patient experienced two). The gastrointestinal obstruction occurred during screening and was unrelated to study procedures. The urinary tract infection occurred in the setting of persistent diarrhea, ultimately leading to intensive care unit admission and treatment discontinuation. The patient with diabetic ketoacidosis was admitted due to poor compliance with insulin treatments and had a complicated hospital course with severe acidosis, changes to glucose and potassium levels, dehydration, and chemotherapy-related neutropenia. After a period of treatment in the intensive care unit, the patient made a complete recovery and resumed treatment.

## Discussion

In the HALT-D study of crofelemer for the prevention of CID in patients with HER2-positive BC receiving PH and a taxane, there was no significant difference in the number of patients experiencing ≥ 2 consecutive days of diarrhea between the crofelemer and control arms. It is probable that the selection of this primary endpoint was not the correct choice to adequately determine efficacy of decreasing diarrhea. In the CONTROL study of neratinib-related diarrhea, the primary endpoint was NCI-CTCAE grade ≥ 3 diarrhea. Most patients in the current study experienced any-grade CID for at least 7 days in the 3-week cycle, and many experienced multiple daily episodes (or higher grades) of diarrhea for several days. This makes the selected primary endpoint of ≥ 2 consecutive days insensitive and of limited use in differentiating crofelemer’s effect from that of control. Conversely, there were a number of secondary endpoints that suggested crofelemer benefit. In both treatment cycles, there was a clinically meaningful difference between the crofelemer and control arms in terms of maximum within-cycle ordinal NCI-CTCAE grade diarrhea, which was statistically significant in cycle 2 based on both investigator assessment and PROs. The odds of having watery diarrhea during cycle 1 was 23% lower in the crofelemer arm. Patients in the crofelemer arm were 1.8 times more likely to see their diarrhea resolve than patients in the control arm in cycle 2.

Use of BTAD was permitted on an as-needed basis; there were no significant differences reported between the arms. Pharmacogenomic studies indicate that specific *ABCB1* genotype variations have an impact in the plasma concentrations of loperamide [29] and opioids in general [30], which could explain why some patients try to maintain loperamide use, while others may disregard it for lack of efficacy. Similarly, FACIT-D scores did not demonstrate significant differences between treatment groups, which may have been related to the study duration. Patients may not have had adequate exposure to crofelemer to assess potential impact on their quality-of-life scores.

Cancer treatment-related diarrhea management has been “reactive” and few “prophylactic/proactive” approaches have been evaluated. CONTROL [31] required neratinib dose escalation with loperamide to achieve the therapeutic neratinib dose. Patients received rescue budesonide and colestipol, plus dietary recommendations, due to loperamide prophylaxis’ inadequacy in controlling neratinib-induced diarrhea. One arm showed improved tolerability when the neratinib dose was escalated during the first 15 days of therapy. Furthermore, the dose escalation schema of neratinib exposes the patient to sub-therapeutic doses and hence inadequate tumor suppression and/or resistance (to neratinib and potentially other similar agents). Hence, a mechanistically appropriate drug was evaluated in HALT-D to ensure that the loading doses of H and P could be administered, followed by maintenance doses.

THP with paclitaxel and crofelemer was more often associated with no diarrhea than the docetaxel-containing regimens and crofelemer (THP/TCHP). Other studies have shown that diarrhea is less frequent with paclitaxel [32, 33]; however, small numbers in HALT-D may preclude definitive conclusions regarding an interaction and further study would be needed.

The limitations of HALT-D include the selected primary endpoint for this population and treatment regimen. There is a lack of uniformity and agreement on how to assess CID; clinical practice guidelines continue to provide recommendations for treatment only [25, 34]. For that reason, HALT-D evaluated a number of measures of CID, and a decision was made to select the incidence of any-grade diarrhea for ≥ 2 consecutive days, assessed by NCI-CTCAE v4.0, as the primary endpoint. Several other measures were assessed with the intent to provide a comprehensive evaluation of, and to enable discussion of, potential CID endpoints for clinical trials. Another limitation was the short and late exposure to crofelemer. Crofelemer was only administered during the first 2/3 cycles of chemotherapy/HER2-targeted therapy. In terms of timing, the first dose of crofelemer was administered orally at the infusion center, 30 min prior to the first chemotherapy/HER2-targeted therapy dose. This may have been too late to better counteract the effects of IV chemotherapy and anti-HER2 therapies on luminal ion channels, as well as to provide the desired protection to the bowel. In addition, loading doses of P and H were administered at cycle 1, per standard of care.

The strength of HALT-D is it being the first of its kind, to our knowledge, to evaluate the prophylactic use of a novel antidiarrheal drug for preventing or mitigating CID in BC (activated charcoal and budesonide have been shown to possibly mitigate irinotecan-induced diarrhea and reduce loperamide use in small colorectal cancer studies [35, 36]; and a review published in 2019 after HALT-D began highlighted minimal success of prophylaxis with agents available at the time [13]). Since currently used antimotility drugs (loperamide, diphenoxylate/atropine) do not target the CID mechanism, HALT-D evaluated a new paradigm for CID management with crofelemer (a potential targeted therapy). In addition to the new approach for prophylaxis of CID, this is the first study to our knowledge that incorporated PROs to assess incidence and severity of loose/watery stools as a continuous variable in patients with BC. Although HALT-D did not meet its primary endpoint for the reasons outlined above, it still has clinical relevance because the data add to literature on how to evaluate diarrhea in a clinical trial setting. Diarrhea is an important and debilitating side effect of HER2-targeted therapies, and HALT-D helps to better understand how to assess diarrhea interventions in future. HALT-D also represents real-world evidence wherein the patient manages their CID in between cycles of treatments. Finally, data collected by patients themselves were comprehensive in terms of recording and providing the information about their experience during treatment. This allowed extensive collection of prospective daily information for the entire duration of the study, as well as objective analyses of multiple CID endpoints as described.

Future directions include the ongoing phase III, double-blind, placebo-controlled real-world evidence OnTARGET study (NCT04538625), which is evaluating crofelemer for the prophylaxis of diarrhea in adult patients with solid tumors receiving targeted therapy agents with or without chemotherapy. The OnTARGET endpoint integrates various characteristics of targeted therapy-associated diarrhea, including time to onset, duration, and resolution of diarrhea, and the possibility that the diarrhea may be cyclical or intermittent in patients receiving cycles of chemotherapy. It is not a binary endpoint that can be easily reached with highly diarrheagenic treatment regimens. OnTARGET is larger than HALT-D, has longer follow-up, and takes into consideration several elements of diarrhea that HALT-D’s primary endpoint was not able to.

## Conclusion

The HALT-D study demonstrated that crofelemer reduces the incidence and severity of grade ≥ 2 CID associated with treatments containing PH and a taxane, especially during the second cycle of TCHP or THP treatment. Furthermore, a significantly larger number of patients in the crofelemer arm versus the control arm either had no loose/watery stools or < 3 loose/watery stools (i.e., grade 1 CID) during the second cycle of chemotherapy/HER2-targeted therapy. Patients in the crofelemer arm were also more likely to see their diarrhea resolve compared with those patients receiving only standard-of-care antidiarrheal medications. The findings of the HALT-D study support further testing of crofelemer in CID.

## Supplementary Information

Below is the link to the electronic supplementary material.

Supplementary file1 (DOCX 40 KB)

## Data Availability

The datasets generated and/or analyzed during the current study are not publicly available due to Protected Health Information regulations, but are available from the corresponding author on reasonable request.

## Abbreviations

AE: Adverse event
BC: Breast cancer
BID: Twice a day
BSFS: Bristol Stool Form Scale
BTAD: Breakthrough antidiarrheal medication
C: Carboplatin
CaCC: Calcium-activated chloride channels
CFTR: Cystic fibrosis transmembrane conductance regulator
CI: Confidence interval
CID: Chemotherapy-induced diarrhea
FACIT-D: Functional Assessment of Chronic Illness Therapy for Patients with Diarrhea
FACT-G: Functional Assessment of Cancer Therapy—General
H: Trastuzumab
HR: Hazard ratio
IV: Intravenously
LRTs: Likelihood ratio tests
NCI-CTCAE: National Cancer Institute’s Common Terminology Criteria for Adverse Events
OR: Odds ratio
P: Pertuzumab
PROs: Patient-reported outcomes
qXw: Every X weeks
SD: Standard deviation
T: Taxane
