# Supplementary material for: HALT-D: a randomized open-label phase II study of crofelemer for the prevention of chemotherapy-induced diarrhea in patients with HER2-positive breast cancer receiving trastuzumab, pertuzumab, and a taxane
Source: Breast Cancer Res Treat. 2022 Oct 25;196(3):571–81. doi: 10.1007/s10549-022-06743-9 (PMC9633499; doi:10.1007/s10549-022-06743-9)
Supplement: Supplementary file 1 — Supplementary file1 (DOCX 40 KB) [file 10549_2022_6743_MOESM1_ESM.docx]

HALT-D: a randomized open-label phase II study of crofelemer for the prevention of chemotherapy-induced diarrhea in patients with HER2-positive breast cancer receiving trastuzumab, pertuzumab, and a taxane

**Author information**
Paula R. Pohlmann **·** Deena Graham **·** Tianmin Wu **·** Yvonne Ottaviano **·**Mahsa Mohebtash **·** Shweta Kurian **·** Donna McNamara **·** Filipa Lynce **·** Robert Warren **·** Asma Dilawari **·** Suman Rao **·** Candace Mainor **·** Nicole Swanson **·** Ming Tan **·**Claudine Isaacs **·** Sandra M. Swain

**Corresponding author:**

Sandra M. Swain, MD, FACP, FASCO

Associate Dean for Research Development

Professor of Medicine

Georgetown University Medical Center

4000 Reservoir Road NW

120 Building D

Washington, DC 20057

Tel: 202-687-4600

Email: [sandra.swain@georgetown.edu](mailto:sandra.swain@georgetown.edu)

**Online resource 1.** FACIT-D scores.

|  | ***n*** | **Median  (min, max)** | **Mean (SD)** | ***p* value** |
| --- | --- | --- | --- | --- |
| **Cycle 1** | | | | |
| **Diarrhea subscale** | | | | |
| Crofelemer | 26 | 43.0 (21.0, 44.0) | 40.5 (6.2) | 0.565 |
| Control | 25 | 42.0 (24.0, 44.0) | 39.8 (6.3) |  |
| **FACIT-D Trial Outcome Index** | | | | |
| Crofelemer | 26 | 85.0 (55.0, 100.0) | 85.4 (10.9) | 0.699 |
| Control | 25 | 86.5 (52.0, 100.0) | 82.8 (14.8) |  |
| **FACT-G total score** | | | | |
| Crofelemer | 26 | 89.0 (57.0, 106.0) | 88.5 (12.9) | 0.37 |
| Control | 25 | 85.0 (49.0, 108.0) | 84.0 (16.5) |  |
| **FACIT-D total score** | | | | |
| Crofelemer | 26 | 131.0 (97.0, 150.0) | 129.0 (14.3) | 0.423 |
| Control | 25 | 127.5 (81.0, 152.0) | 123.8 (20.4) |  |
| **Emotional wellbeing** | | | | |
| Crofelemer | 26 | 19.0 (9.0, 23.0) | 18.1 (3.8) | 0.427 |
| Control | 25 | 17.0 (4.0, 24.0) | 17.3 (4.4) |  |
| **Functional wellbeing** | | | | |
| Crofelemer | 26 | 20.5 (8.0, 28.0) | 21.1 (5.8) | 0.636 |
| Control | 25 | 20.0 (6.0, 28.0) | 20.0 (6.6) |  |
| **Cycle 2** | | | | |
| **Diarrhea subscale** | | | | |
| Crofelemer | 25 | 38.0 (22.0, 44.0) | 34.9 (6.8) | 0.885 |
| Control | 23 | 35.0 (16.5, 44.0) | 34.1 (7.8) |  |
| **FACIT-D Trial Outcome Index** | | | | |
| Crofelemer | 25 | 77.2 (46.0, 93.0) | 73.7 (14.9) | 0.332 |
| Control | 23 | 68.0 (29.8, 98.0) | 68.7 (18.1) |  |
| **FACT-G total score** | | | | |
| Crofelemer | 25 | 86.0 (43.2, 107.0) | 81.8 (16.7) | 0.445 |
| Control | 23 | 77.0 (46.8, 104.0) | 77.3 (19.3) |  |
| **FACIT-D total score** | | | | |
| Crofelemer | 25 | 121.2 (73.2, 143.0) | 116.7 (19.8) | 0.386 |
| Control | 23 | 110.0 (63.3, 147.0) | 111.4 (23.3) |  |
| **Emotional wellbeing** | | | | |
| Crofelemer | 25 | 19.0 (5.0, 24.0) | 18.4 (4.5) | 0.860 |
| Control | 23 | 18.0 (6.0, 24.0) | 18.7 (4.7) |  |
| **Functional wellbeing** | | | | |
| Crofelemer | 25 | 20.0 (7.0, 28.0) | 18.7 (6.5) | 0.336 |
| Control | 23 | 15.0 (3.0, 28.0) | 16.6 (7.5) |  |
| **Cycle 2 versus cycle 3; crofelemer arm only** | | | | |
| **Diarrhea subscale** | | | | |
| Cycle 2 | 24 | 37.5 (22.0, 44.0) | 34.6 (6.9) | 0.784 |
| Cycle 3 | 24 | 36.5 (13.0, 44.0) | 34.3 (9.1) |  |
| **FACIT-D Trial Outcome Index** | | | | |
| Cycle 2 | 24 | 74.6 (46.0, 93.0) | 73.2 (15.0) | 0.563 |
| Cycle 3 | 24 | 81.5 (31.7, 100.0) | 74.2 (18.0) |  |
| **FACT-G total score** | | | | |
| Cycle 2 | 24 | 84.9 (43.2, 107.0) | 81.5 (17.0) | 0.383 |
| Cycle 3 | 24 | 85.0 (46.0, 108.0) | 83.2 (17.1) |  |
| **FACIT-D total score** | | | | |
| Cycle 2 | 24 | 119.1 (73.2, 143.0) | 116.1 (20.0) | 0.516 |
| Cycle 3 | 24 | 123.0 (64.7, 152.0) | 117.5 (23.3) |  |

*FACIT-D* Functional Assessment of Chronic Illness Therapy for Patients With Diarrhea, *FACT-G* Functional Assessment of Cancer Therapy—General, *SD* standard deviation

**Online resource 2.** Medians and percentage reductions in median watery bowel movements (Bristol Stool Form Scale 6–7) from control by week in cycles 1, 2, and 3. Yellow highlighted cells: favors Crofelemer; red, favors control.

|  | **Cycle total** | **Week 1** | **Week 2** | **Week 3** |
| --- | --- | --- | --- | --- |
| **Cycle 1** | | | | |
| Crofelemer, median (range) | 14.5  (0, 102.0) | 5.5  (0, 19.0) | 6.0 (0, 43.0) | 2.0 (0, 40.0) |
| Control, median (range) | 19.0  (4.0, 72.0) | 5.0 (0, 23.0) | 8.0 0, 33.0) | 4.0 (0, 16.0) |
| Absolute difference from control | –4.5 | 0.5 | –2.0 | –2.0 |
| % reduction from control | 23.7 | –10.0 | 25.0 | 50.0 |
| *p* value (Wilcoxon rank sum test) | 0.2834 | 0.6363 | 0.6122 | 0.1915 |
| **Cycle 2** | | | | |
| Crofelemer, median (range) | 14.0 (0, 139.0) | 4.0 (0, 48.0) | 7.0 (0, 51.0) | 4.0 (0, 40.0) |
| Control, median (range) | 17.5 (1.0, 75.0) | 6.0 (0, 22.0) | 8.5 (0. 29.0) | 3.5 (0, 34.0) |
| Absolute difference from control | –3.5 | –2.0 | –1.5 | 0.5 |
| % reduction from control | 20.0 | 33.3 | 17.6 | –14.3 |
| *p* value (Wilcoxon rank sum test) | 0.4076 | 0.5876 | 0.3171 | 0.8194 |
| **Cycle 3** | | | | |
| Crofelemer, median (range) | 19.0 (2.0, 43.0) | 5.5 (0, 14.0) | 7.0 (0, 22.0) | 4.0 (0, 16.0) |
| Control, median (range) | 14.0 (0, 59.0) | 5.0 (0, 17.0) | 5.0 (0, 29.0) | 2.0 (0, 15.0) |
| Absolute difference from control | 5.0 | 0.5 | 2.0 | 2.0 |
| % reduction from control | –35.7 | –10.0 | –40.0 | –100 |
| *p* value (Wilcoxon rank sum test) | 0.1358 | 0.5194 | 0.2429 | 0.1430 |
| **Crofelemer group: cycle 2 vs 3 (only analyzing paired data)** | | | | |
| Cycle 2, median (range) | 13.0  (0, 139.0) | 4.5 (0, 48.0) | 6.5 (0, 51.0) | 3.0 (0, 40.0) |
| Cycle 3, median (range) | 19.0 (2.0, 43.0) | 5.5 (0, 14.0) | 7.0 (0, 22.0) | 4.0 (0, 16.0) |
| *p* value (Wilcoxon signed rank test) | 0.1535 | 0.1636 | 0.3800 | 0.5049 |

**Online resource 3.** Non-diarrhea AEs in > 10% of patients in either arm during cycles 1 and 2.

| **Patients with  ≥ 1 AE** | **Crofelemer (*n*=26)** | **Control (*n*=25)** |
| --- | --- | --- |
| Fatigue | 10 (38.5) | 9 (36.0) |
| Nausea | 10 (38.5) | 9 (36.0) |
| Anemia | 5 (19.2) | 2 (8.0) |
| Anorexia | 4 (15.4) | 3 (12.0) |
| Mucositis oral | 4 (15.4) | 3 (12.0) |
| Constipation | 4 (15.4) | 2 (8.0) |
| Gastroesophageal  reflux disease | 4 (15.4) | 1 (4.0) |
| Myalgia | 3 (11.5) | 2 (8.0) |
| Rash maculo-papular | 3 (11.5) | 2 (8.0) |
| Hypokalemia | 3 (11.5) | 1 (4.0) |
| Rash acneiform | 3 (11.5) | 1 (4.0) |
| Vomiting | 3 (11.5) | 1 (4.0) |
| Anxiety | 3 (11.5) | 0 |

*AE* adverse event

Data are number of patients, *n* (%)

All AEs shown were grade 1/2 except nausea, where there was one grade 3/4 event in the control arm
